# Covid-19 and tracing methodologies: A lesson for the future society

**DOI:** 10.1007/s12553-021-00575-1

**Published:** 2021-08-07

**Authors:** Teresa Scantamburlo, Atia Cortés, Pierre Dewitte, Daphné Van der Eycken, Ralf De Wolf, Marijn Martens

**Affiliations:** 1 European Centre for Living Technology, Ca’ Foscari University of Venice, Venice, Italy; 2 Barcelona Supercomputing Center, Spain; 3 KU Leuven Centre for IT & IP Law, Katholieke Universiteit Leuven, Leuven, Belgium; 4 Imec-Mict-UGent, Ghent University, Ghent, Belgium

**Keywords:** Coronavirus, Contact tracing methodologies, Social impact, Privacy

## Abstract

As the new *coronavirus* (*SARS*-CoV-2) surged across the globe, new technical solutions have supported policy makers and health authorities to plan and modulate containment measures. The introduction of these solutions provoked a large debate which has focused on risks for privacy and data protection. In this paper we offer an analysis of the available technical approaches and provide new arguments to move beyond the ongoing discussions. In particular, we argue that the past debate missed the opportunity to highlight the societal aspects of privacy and to stimulate a broader reflection on the actions needed to serve the good of society. With this paper, as well as providing an accessible review of the technical and legal aspects of the proposed solutions, we aim to offer new stimuli to reconsider contact tracing and its role in helping countries navigate the current pandemic.

## Introduction

Since the beginning of the COVID-19 pandemic, the computer science community has been contributing ideas and practical solutions to tackle this global crisis. Significant efforts have been directed towards developing digital contact tracing applications to complement lockdown measures and, ultimately, curb the spread of the virus. In general, the goal of these apps is to notify people who have recently been in contact with a person diagnosed positive, and to provide them with guidance on how to proceed to avoid the further spreading of the disease, such as observing the quarantine period and getting in touch with public health authorities.

Proposals of this sort abound in literature and vary in several respects including the technology employed. Contact tracing has already been used in past epidemic diseases such as the Ebola virus [1]. Traditionally, it is conducted manually by human interviewers. Because of the magnitude of COVID-19, more recent methods rely on mobile applications and attempt to improve the extent and the efficiency of data collection and retrieval.

In the spectrum of tracing methodologies, the role of digital technology can vary significantly. At one extreme, the application only supports the work of human interviewers replacing the paper forms [2]. At the other extreme, the system, infused with learning capabilities, acts as a well-informed orchestrator making risk predictions and recommendations at personal level [3].

This variety is not only a matter of technological diversity but also a societal dilemma. Indeed, a more effective tracking process, enabled by mobile technology’s pervasiveness, can also translate into privacy breaches and, at worst, systematic forms of societal control.^1^ These possibilities raised several concerns about the practical effectiveness and the privacy guarantees of these apps. Also, many organisations put forward guidelines and principles for the design and deployment of digital contact tracing apps following data protection legislation and human rights (European Commission [4] and European Data Protection Board [5]).

In this paper we argue that the discussion was framed too narrowly in terms of technical requirements and competing architectures. An example of this narrow focus is given by the polarized discussion between the centralised vs. decentralised approaches (see Sect. 2.2.), which differ in the way they generate the identifiers needed for the system to properly work, share information between devices and compute the risk score for each individual. We believe that this and similar debates, albeit essential, distracted from a broader reflection on the needed actions to put the tracing process to service for the good of society, not only for the contingency of a large-scale crisis.

We first provide an overview of three different contact tracing methodologies and suggest to what extent humans and machines can (co)operate. We then survey the main principles put forward in legal literature and discuss some limitations which affect the ongoing discussions. Finally, we suggest three arguments highlighting the importance of the societal context and human oversight.

## Tracing methodologies

Contact tracing methodologies were already in use over 500 years ago to control the great pox (also known as syphilis) when a group of Italian doctors started investigating the spread of the disease in the search for the “patient zero”.^2^ There are several examples over the history of medicine, from AIDS to Ebola, where tracing methods were implemented to identify symptomatic individuals and, when needed, apply strategies of isolation. The societal and ethical concerns raised by such techniques at the time are still largely present today. Those include the fear of disclosing personal information on our societal interactions, the lack of trust in the public institution tasked with the collection and further processing of the said data, the potential for discrimination and stigmatisation and the necessity to partially bypass the democratic debate due to the urgency of the situation. The use and the efficacy of digital technologies for tracking and curbing the spread of the virus are still under review and different institutions are monitoring their introduction around the world.^3^

In this section we provide our own classification which aims at suggesting different levels of human and machine computation. On the one hand, there are methods which rest on the human capability to collect information through interviews or self-reports. These have already been in use in past pandemic diseases and can also exploit digital devices to support and improve data collection tasks of health professionals. On the other hand, there are methods using technology to warn users of a potential exposure either in the form of a binary signal (e.g. “being in contact with a positive case or not”) or a risk score. We are aware that our taxonomy is all but exhaustive and, in certain respects, it may even be disputable,^4^ but our goal is not to cover the plethora of all existing applications. Instead, the primary purpose is to suggest the continuity between digital solutions and human tracing and, secondly, to highlight the role played by machines and the different types of human–machine interaction. In other cases, the machine could be a silent medium replacing pen and paper, in other cases, the machine can be more active and sends alerts to the user automatically, often after a given consent. We call this second type of application “machine-driven”^5^ since the technological element takes an active role in eliciting a desired course of actions based on a “simple” warning alert or a risk prediction and personalised messages. Technically speaking, what is automated here is the notification process or the prediction of infection and humans can still take care of other important activities (e.g. instructing users who have been notified), but the human intervention is somehow dependent on the tech layer. Table 1 summarises the main benefits and limitations of each contact-tracing method that is described in the following subsections.

**Table 1:** Benefits and limitations con contact-tracing methods

Contact-tracing method	Benefits	Practical limitations	Ethical limitations
Human	-Well-known protocol, used in previous epidemiological crisis -Notifications are made by other humans	-Requires human infrastructure to guarantee that the social environment of an infected person is properly followed-up -Accuracy relies on the contacts provided by the infected person, which might be incomplete	-Collection of personal information of contacts -Could create stigmatisation towards vulnerable groups
Location/Proximity	-Increases the ability to collect data and find possible social interactions with infected people -Privacy-preserving approach should encourage citizens to use the app and increase its efficiency	-Technology is sensible to environmental barriers (walls, windows) which might lead to false positives -Accuracy also relies on the number of people using these apps	-Automated notifications without human supervision might have a negative impact on human acceptance -Lack of trust in the technology and the use of the data -Feeling of surveillance and fear of law enforcement
AI-based	-In addition to binary outcome infection, solutions can offer levels of risk of infection based on the kind of interaction or forecast spread of disease by combining and analysing different sources of information	-Data interoperability among different agencies and countries could be challenging	-Techno-solutionism increases social inequality by not guaranteeing the same access to information to vulnerable groups -Environmental impact of data collection and AI model training

### Human-driven tracing

Traditionally, contact tracing has been handled through personal interviews between health professionals and patients. The aim is to identify possible contacts of the infected person and monitor them for several days after the notification of infection. Protocols need to be put in place as soon as the case is confirmed to be effective, although they may vary among countries and viruses. In the case of COVID-19, health agencies try to identify contacts where transmission could have happened (e.g. interactions longer than 15 min and within a distance of 2 m over the last 14 days prior to the positive result). This allows to elaborate on the list of personal contacts, albeit it risks to be very imprecise as they rely on the imperfect recollection of persons interviewed as well as their criteria to measure if a contact was significant enough to be considered as a random contact or as a case to be analysed. Some studies (e.g., Ferretti et al. [6] suggest that this technique is not sufficient to control a pandemic such as COVID-19 and the individuals at risk of transmission.

Manual contact tracing can also make use of mobile applications. For example, in 2016 a group of researchers proposed a software to improve data collection and storage for Tuberculosis tracing in Botswana [2]. The intended users are health care workers who need to operate in settings with limited resources, and the interaction with the patients is still guided by humans. In the early days of the COVID-19 pandemic the technological intermediation moved towards more distributed forms of data collection. For example, several governments, research centres and institutions created online survey forms to gather health information from self-reports. The common structure of these surveys starts by asking for some personal information: gender, age range and location (the level of detail depends on each survey and country, going from the name of the city, to the zip code or even the street name), but could also be extended to the professional sector, level of income and others. Next, a series of questions were asked to reveal possible COVID-19 symptoms. All this information is collected and analysed in order to study the evolution of the pandemic and identify those areas where the pandemic was more active, and ultimately to help governments and health authorities to make decisions. Note that, while these tools are offered to large populations (not just a group of professionals), the interactions with user is limited to (voluntary) self-reporting and not intended to deliver specific messages or recommendations.^6^

### Machine-driven contact tracing

For decades, economic, health or environmental emergencies have led to crisis-driven innovation. In the case of COVID-19 crisis, one of the main topics of discussion has been the digital contact tracing methodologies, generally deployed in mobile apps. While in human-driven methodologies people are notified by other people, with machine-driven approaches the notification mechanism is automated. On the one hand, this feature can significantly reduce costs in terms of time and people needed to deal with large pandemics. On the other, the active intermediation by digital tools in tracing processes can introduce further privacy and surveillance concerns (see Sect. 3). In addition, since people can have different reactions depending on the nature of the intermediation (i.e. human or technological), we may expect that, in the long run, different notification mechanisms can generate distinct behavioural patterns within the population. For example, machine-driven solutions, and in particular those incorporating learning capabilities, could favour the initiative and the autonomy of individual users with respect to actions to be taken after an exposure notification and, as a consequence, increase chaos in associated services (e.g. when a large number of users contacts health operators simultaneously these may not be able to serve all requests).

#### Location-based and proximity-based solutions

The main purpose of contact tracing apps is to reduce the spread of the pandemic and support policy makers in planning alternatives to stringent interventions such as lockdown measures. Indeed, lockdowns are not sustainable in the long term and cause significant impacts in our daily lives, both from an economic and societal perspective. The objective of digital contact tracing is to monitor contacts among citizens and identify those at risk of being contagious. This methodology is designed to help governments and health authorities in making decisions more efficiently by sending prompt alerts to people who were in contact with a confirmed case and applying selective measures like isolation. The reaction time in these situations is crucial to tackle the contagion rate and avoid the spreading of the virus.

We can differentiate between two prevailing means of digital contact tracing. The first one uses a location tracing methodology with GPS or network-based location tracking.^7^ This option has been ruled out in many countries across the EU since, according to the recent Guidelines of the (European Data Protection Board [5], other less privacy-invasive can achieve the same goal. With respect to this option, the main concern regards privacy since both GPS and network-based solutions can be active 24/7 in users’ phones. Also, they can collect more data than strictly necessary to check whether an encounter could lead to an infection and informing the concerned persons of such risk. Another critical aspect regards network-based solution which can do not require active user’s participation (i.e. download and installation), thereby guaranteeing penetration. To the best of our knowledge, Israel, Iran, Cyprus, China, Indonesia, Bulgaria and Ghana have made use of location-based solutions.

The second option is based on proximity data usually collected via Bluetooth Low Energy (BLE), a technology used to transfer data from one device to another, mostly over a short distance. BLE is most commonly used to connect peripherals (e.g. headphones) to devices like smartphones and is omnipresent in almost all modern mobile devices and thus accessible to a large number of people. The characteristics of BLE against other technologies are (i) low power consumption, allowing contact tracing apps to run for hours without draining mobiles' batteries too fast and (ii) indoor operation as a short-distance tracker. However, BLE is also sensitive to false positives as proximity estimation does not always detect architectural obstructions between two individuals that have been identified as exposed [7].

The EDPB has promoted proximity-based solutions as they adopt a privacy-preserving design following the basic principles of the General Data Protection Regulation (GDPR), such as data minimisation and purpose limitation. Among these solutions, two types of protocols have been proposed: (i) centralised solutions such as the ROBERT protocol (that store the data in a central server [8, 9] and (ii) decentralised options such as the DP-3 T protocol that ensures that personal data and computation stays entirely on the user’s phone [10, 11]. The main purpose is to help epidemiologists to build the network of contacted people that are potentially infected. No other personal or health information is collected or required. The DP-3 T protocol enhances user control by giving them a choice to voluntarily share the information gathered by their mobile devices with health authorities. Note that the DP-3 T solution is more properly acknowledged to be an exposure notification app since contact data are stored in the user’s phone.

#### AI-based solutions

Another option to track and limit the spread of the virus is to incorporate Artificial Intelligence into tracing applications. Note that the role played by AI in the control of the COVID-19 pandemic is considerable and not limited to tracing applications. Significant efforts went to diagnosis from medical imaging or voice analysis,^8^ drug discoveries and societal simulations (see [12] for a review). Here we will focus on AI solutions for tracing purposes.^9^

Usually AI-based tracing applications allow to infer knowledge about the risk of infection and the spread of the virus in a geographical area. A proposal going in this direction came from Yoshua Bengio’s team (at Mila, Canada) with the so-called COVI app, whose main functions are: (i) to inform individuals of their infection risks and (ii) to support governments in better understanding the disease transmission and planning containment policies [3]. Another mobile application proposes to use an AI algorithm for classifying users into four classes (no risk, minimal risk, moderate risk and high-risk) and sending an alert for check health recommendation to both the users and health departments [13].

An important distinction between location- and proximity-based apps (such as those described in Sect. 2.2.2) and AI-based solutions is the type of information sent to the user. Instead of sending binary information about whether a user has been in contact with an infected person or not, AI-based applications inform the user of the risk of infection through the aggregation of thousands of data points. For example, COVI sends a message reflecting the probability that the person has been infected and recommending a specific course of actions. The prediction of the risk of infection is enabled by a machine learning model combining different information sources regarding both users’ individual profile (e.g. demographic, existing health issues and presence of new symptoms) and users’ interaction, based on Bluetooth proximity detection. The machine learning model computes users’ current and past contagiousness (their risk level) locally. When two phones with the app meet, they exchange information about each other’s risk. As the app accumulates information the risk estimated is revised and, if a revision is sufficiently important, an updated message is sent to its relevant contact.

Some scholars claim that the introduction of AI and, in particular, machine learning models into tracing applications can help detect the early signals of the disease before they propagate throughout the population [3, 14]. This argument is also reinforced by the lack of human tracers, whose number turns out to be insufficient to interview high volumes of positive cases and find out new potential infections. For example, a study claimed that last year in England “tracers typically reached less than half of the close contacts of people who’d had a positive COVID-19 test” [15].

In general, although their use is meant to support and complement manual tracing, AI-based applications are proposed to offer a greater automation level reducing human efforts in the early phases of the pandemic where symptoms are either absent or not clearly discernible [3]. Also, the proponents of AI-based solutions claim that, by offering risk predictions and customised recommendations, these apps promise to empower individuals with knowledge to protect themselves and take preventive measures [3]. To increase public trust, moreover, AI-based solutions can adopt a privacy-protecting approach [3], for example, requesting consent for all collection and use of personal information (see Sect. 3 for a review of data protection requirements). However, despite the positive inspiration motivating AI-based tracing apps, there is still a lack of evidence proving that increased levels of automation meet the expectations of end users and bring them greater empowerment. In addition to the final social impact, it is also necessary to take into account other dimensions to assess the benefits of deploying such solutions, such as the environmental impact related to the cost of storing huge amounts of data required to train AI models. As we will see in Sect. 4, design specifications can misalign with how users concretely approach and use a tracing application and this situation can lead an app to fail.

## Legal implications of contact tracing for COVID-19

As the number of digital contact tracing applications increased over the past months, a lively debate took place on the impact that such solutions could have on individuals’ fundamental rights to privacy, data protection, health and non-discrimination.^10^ At the European level, this debate translated into concrete questions about the legal acceptability of machine-driven tracing applications and their compliance with the European regulatory framework. Does the General Data Protection Regulation (GDPR) apply in this context? What principles should guide the design of these apps? And what actions follow from them?

To verify whether digital contact tracing apps fall within the material scope of application of the GDPR, it is necessary to check whether they involve the ‘processing’ (Art. 4(2) GDPR) of ‘personal data’, i.e. any information relating to an identifiable natural person (Art. 4(1) GDPR). Regardless of the technology used to perform contact tracing, the consensus is that they do. While it is rather obvious for contact tracing based on geolocation data – the privacy-invasive and repurposing potential of which is well-documented [16, 17] – the same is true for BLE-based solutions, deemed as the most privacy-preserving alternatives. As highlighted in the privacy analysis of the DP-3 T protocol, even decentralised options based on the sharing of emitted EphIDs are vulnerable to re-identification attacks and, therefore, would warrant the qualification of the data processed as ‘personal’ [10, 11]. Without delving into the intricacies of the ‘identifiability’ threshold under Art. 4(1) GDPR, one can reasonably assume that contact tracing apps will fall under the scope of application of the GDPR and, as such, will need to abide by the various principles and rules prescribed therein.

Another fundamental task is to identify and adequately qualify the actors involved in the processing operations, as this will determine the allocation of responsibility, liability and accountability under the Regulation. Under the GDPR, that entity is the ‘controller’, i.e. the one determining the ‘purposes’ and the ‘means’ of the processing activities. While various options can be considered – involving both public and private actors –, the European Commission recommends a model where such responsibility would fall to national health authorities, or the entity carrying out tasks of public interest in the field of health. This, underlined by the (European Commission [4]) and the (European Data Protection Board [5]), is essential to foster public trust and guarantee sufficient adoption.

Besides the applicability of the GDPR, it is to be noted that the data produced via the smart devices are also protected under the ePrivacy Directive, which prescribes that storing information on a user’s device or gaining access to information already stored is allowed only with the consent of the user or if the storage and/or access is strictly necessary for the app installed or activated by the user (Article 5(3)). In the same vein, location data can only be transmitted to authorities or other third parties if they have been anonymised or, for data indicating the geographic position of the terminal equipment, with the prior consent of the users (Article 9(1)).

Operators of mobile apps offering contact tracing functionalities will need to follow a security and data protection by design approach. Table 2 summarises some of the most important principles and rules laid down in the GDPR and ePrivacy that should be taken into account to develop, design, select and use applications that are based on the processing of personal data.

**Table 2:** GDPR and ePrivacy Directive best practices for contact-tracing solutions

Principles	Definitions	Best Practices
Access to terminal equipment	Article 5(3) ePrivacy Directive requires either (i) the user’s freely given, specific, informed and unambiguous consent or (ii) to justify that the storage and access is strictly necessary to ensure the proper functioning of a service explicitly requested by the user	In the context of contact tracing apps, it could be argued that access to terminal equipment is strictly necessary for the functioning of BLE-based digital contact tracing solutions
Lawfulness	Article 5(1)a GDPR requires controllers to justify personal data processing using one of the lawful grounds listed in Article 6(1)	As suggested by the EDPB, the appropriate lawful ground would be, in most cases, Article 6(1)e (task carried out in the public interest)
Special categories of personal data	Article 9(1) prohibits the processing of special categories of personal data unless one of the exemptions listed in Article 9(2) applies	As suggested by the EDPB, the relevant exemption would be, in most cases, Article 9(2)i (reasons of public interest in the area of public health) or h (preventive or occupational medicine)
Transparency	Articles 12, 13 and 14 GDPR require controllers to report about their processing activities in a concise, transparent, intelligible and easily accessible form, using clear and plain language	-Provide the identity and contact details of the controller, the purposes and lawful ground of the processing, the recipients of personal data if any, the retention period and the existence of the multiple prerogatives granted to data subjects such as the right to access and erasure -Transparent and verifiable development through open-source code, external audits and publicly available Data Protection Impact Assessments
Purpose limitation	Article 5(1)b GDPR requires personal data to be (i) collected for explicit, specified and legitimate purposes, i.e. purpose specification, and (ii) not further processed in a manner that is incompatible with those purposes, i.e. compatibility assessment	-Only collect personal data the repurposing potential of which is limited, such as ephemeral identifiers -Avoid the bundling of functionalities within the same app (e.g., a single app providing general information, symptom checker features and contact tracing) or grant users granular control over which of them he or she wishes to opt-in to
Data minimisation	Article 5(1)c GDPR Requires controllers to only collect and further process personal data that is necessary to the purposes that have been specified	-Avoid the use of geolocation and/or movement data (BLE is less privacy-invasive) -Avoid storing the exact time of contact or any type of metadata that is not specific to the contact or duration
Storage limitation	Article 5(1)e GDPR Tailor the retention period according to the purposes of the processing	Proximity data should be deleted as soon as they are no longer necessary for alerting individuals (or EphIDs in the case of BLE-based solutions) or any personal data stored in the backend server

## The debate on contact tracing applications: A missed opportunity?

So far, the debate has highlighted important implications of digital tracing applications thereby suggesting that using technological advances to tackle societal issues is laudable but insufficient by itself. Technological intermediation can offer great opportunities in reaching out to large populations and reducing the time for tracing the whole chain of contacts. However, it can also justify invasive practices of data collection and favour surveillance mechanisms for societal control and profiling. Furthermore, if not careful, machine-driven contact tracing methodologies might result in a pure consequentialist and technological deterministic attitude, in which solutionist tech (cfr. [18] is perceived as a necessity in tackling this crisis and privacy treated as good that can be traded rather than a fundamental right.

Contact tracing methodologies should be treated as systems producing value and giving meaning, not merely neutral technical artefacts. They are developed with certain goals in mind and are thus calibrated to reach that specific goal as efficient as possible [19]. The GDPR provides essential safeguards to address privacy and data protection issues (see the principles described in our table) along with other existing legislation. Based on that, several organisations and researchers provided useful guidelines for assessing digital contact tracing apps (e.g. [20]. It could also be argued that the debate, at least in Western European countries, has been mindful of privacy issues and data protection. So, rather than focusing only on the urgency of the crisis and the pressing need to flatten the curve of contagion, there has been room for legal and human rights considerations which have promoted the discussion of fundamental principles such as data minimization, consent and voluntary use. In addition, in various countries it was explicitly described how digital tracing methodologies have a supportive role and will not replace manual tracing efforts, thereby opposing a view in which technology occupies the driver’s seat.

However, the current debates have largely ignored the societal context of these apps. In the following sections we propose three arguments that, in our opinion, move beyond the current debate and offer new stimuli to reconsider contact tracing and its role in helping countries navigate the current pandemic.

### Control, secrecy and appropriateness

First, we argue that the current discussions on privacy and tracing methodologies are too narrowly focused on control and access restriction. Scanning through existing applications and protocols, it is noticeable how two prominent perspectives on privacy are put forward and translated into the design, that is, ‘privacy as secrecy’ and ‘privacy as control’. The former perspective ensures full anonymity, or at least tries to. For example, those who employ GPS or network-based location tracking put forward solutions to anonymize personal data. Others, like the DP-3 T, propose a decentralized design that shifts the processing operations from a central entity to end-users devices. The latter perspective offers control options to regulate and manage their information flow. For example, the exposure notifications systems of Google and Apple allows one to opt in to use exposure notifications after the public health authority app is downloaded. One can also decide, when diagnosed positive to Covid-19, to share random IDs with the application.

Although both secrecy and control are important parts of privacy, they should not be treated as one and the same thing [21]. Rather we want to underline and stress out the importance of a contextual approach to privacy (cfr. Contextual integrity [22, 23]). Contextual integrity (CI) does not focus on privacy expectations in terms of ‘control’ or ‘secrecy’ but in terms of ‘appropriateness’.

According to CI the focus on the control of personal data and increased exposure is only part of the anxiety but limited in itself. In her framework, [23] argues to focus on informational norms (what is appropriate and what is not within and between contexts), that consists of three parameters: actors, information types, and transmission principles. Contextual integrity is achieved when a particular flow, or transmission of information from one party to another is appropriate in terms of the type of information that is shared, the identity of the sender, how it is shared and the receiver of the data. CI moves beyond an individual perspective on privacy and denies a false contradiction between privacy and using personal information for various reasons, including tracking location or monitoring everyday behaviours. If the flow is appropriate (not necessarily in ‘control’ or ‘secret’) then contact tracing does not necessarily reduce privacy expectations.

Contact tracing apps, like other digital services, build upon existing practices and rules which influence design choices more or less implicitly. This means that the elements charactering the context of this technology, such as the actors involved with their tasks and responsibilities, or the types of exchanged information, depend to some extent on the features of administrative routines and protocols operating in an organization. More specific rules can be detailed in national pandemic strategies and applied in particular time of crisis to coordinate and improve efforts. For example, the entity to whom report a positive test or ask advice in case of a possible contagion can be a health agency which operates according to organizational and social norms. These may regard the type of information collected and the communication chains to be followed in a time of pandemic, as well as the commitment to alleviating illness and promoting health. Identifying and understand such norms is a valuable exercise not only to anticipate which patterns of flow can harm people privacy and rights but also to identify responsibilities of actors involved. This would be even more essential when communication flows through an intricate web of connections as those arising in complex institutions (such as national health care services), where decisions are distributed across multiple actors.

Note that the discussion of CI span both human- and machine-driven tracing methodologies since the organizational and social norms governing the flows of information can be independent of the substrate used to provide a service (in our case to notify users at risk of carrying the virus as early as possible). On the one hand, this would challenge the naïve assumption that risks for privacy and discrimination originate only from computer-mediated communication. On the other hand, the design of communication technologies can obfuscate the meaning of certain roles or norms characterizing the context of application. For example, the ownership of the server storing information (for example, think of the centralized versions of exposure notification apps) with the associated powers and duties might be unclear or poorly communicated to the user.

Arguably, the current pandemic is quite unique in its impact and affecting societies at their core. It is difficult to imagine what life will be like, let alone one’s opinion about the usage of surveillance technology and tracing methodologies to limit the spread of Covid-19 and other pandemics. It is therefore necessary to negotiate this relatively new context that we are in to identify practices that defy privacy norms, which requires a shift beyond privacy as control or secrecy.

### Technical specifications versus technology usage

A second issue concerns the misalignment between how technical artifacts work and how end-users imagine these to work. Every individual forms a specific idea of how a technological system works and why it works in a certain way (cf. algorithmic imaginary [24]). It is this algorithmic imagination that fuels how people form opinions and whether or not they want to use a specific system and under what circumstances. Even if only imagined, this image is real in its consequences.

In the context of machine-driven contact tracing, the information people received was, at best, sparse and inconsistent. Up to the date, some countries have designated institutions in charge of controlling personal data related to contact tracing apps.^11^ However, other governments have not clearly communicated yet who would be in charge of the development and management of these applications and the collected data, how data would be processed (Bluetooth vs GPS, AI vs algorithms), or what specific goals would be pursued by the application. There is a significant difference between a contact-tracing app that will merely inform users (i.e., that they may have been in contact with someone that had COVID-19), that enforces quarantine measures, or one that could be accessed and used by governments for purposes other than the containment of the virus’ spread.

In the context of the COVID-19 tracing apps, the general goal is to track the spread and contaminations of the virus. A close physical encounter is considered a risk, thus, when short range technology (i.e., BLE) detects a close proximity of personal devices (i.e., smartphones), the app will interpret this as a risky encounter. These include completely safe contacts (e.g., behind a window or with sufficient precautions). These imperfections could decrease the perceived accuracy of and trust in these systems. Additional concerns would come when dealing more nuanced information such as risk predictions and behavioural recommendations. How would the user interpret a risk measure? Would it make sense of the risk estimates automatically revised by the machine leaning model?

It is not entirely clear who, how, and why exactly these COVID-19 tracing applications will be developed and maintained. Based on this inaccurate and incomplete information, people could imagine and supplement their algorithmic imaginaries with erroneous insights (e.g. the government will use these applications to spy on their citizens). It is then fair to ask not only *what* kind of technology will be used, but also *why* [25] and *how*. These questions go beyond the technical requirements; governments should also justify if they are necessary, proportional, scientifically sound and time-bounded to solve the main problem [26].

### Discussing the impact of design choices

A third and final issue concerns how considerably more importance is attached to the development of contact tracing apps as opposed to discussions on adoption and appropriation. It is crucial to discuss and assess the impact of choices made during development. However, the privacy negotiation process must continue in the deployment stage. Users will evaluate the appropriateness of data flows and adjust their algorithmic imaginary while using the application.

Another important consideration regards the assessment of the effectiveness of the adopted solutions. Among others, this includes the discussion as to whether the applications employed operate as expected. Follow-up studies suggested that certain proximity-based approaches suffer from important technical flaws. For instance, it has been shown that Australia’s app has worked only 25% of the time on some devices because “the Bluetooth “handshake” necessary to register proximity between two phones doesn’t work if the phone screen is locked” [27]. Another study testing the Italian, Swiss and German apps in a tram reported that the technology was very inaccurate and no better than a random notification system [28].

In addition to the comprehensiveness of the data being collected, the ways how users adopt and use contact tracing apps will likely influence the potency of contact tracing apps (e.g. if end-users do not trust the organization storing the data, they could decide to sporadically or indefinitely turn off their Bluetooth/delete the application). Also, mechanisms allowing citizens to provide their feedbacks and flag issues related to data protection or specific app’s functionalities are a fundamental step to promote the adoption of tracing apps across the population. Note that collecting and taking care of citizens’ opinion serves not only an important social function but also a scientific and technological purpose. Solving problems by means of scientific and technical tools is in fact an attractive option, even in social and political domains. However, the adoption of tech solutions for solving social and policy problems is exposed to several ideologies (e.g. tech solutionism) and the seduction of quantification especially in times of global uncertainties [29]. So, it is critical that tech-based solutions, such as contact tracing apps, once deployed, keep being tested by experts and open to public opinion so as to collect a spectrum of observations as larger as possible. Regular testing with participatory assessment practices^12^ (e.g. through citizens’ assemblies and public deliberation) would contribute to create better narratives of our tech solutions and more elements to exercise trust or distrust in this technology.

## Concluding remarks

In this paper we gave an overview of the technology proposed to control the spread of the COVID-19 pandemic and the public debates that originated from it. Our discussion suggested that past and present discussions could miss an important opportunity. The COVID-19 pandemic gives many stimuli to think about how a global crisis can be tackled through a large technological infrastructure. Although many countries moved towards privacy-friendly solutions, such as the DP-3 T protocol, the effect of these notification mechanisms on the whole population is still unexplored: do the technological intermediation achieve the intended goal? Does it work as expected? What is the impact of such technology in the long run? These simple questions point to significant technical and societal considerations that are essential to investigate the effectiveness of the chosen technology. For example, it has been suggested that proximity-based apps need a high level of adoption to be effective for decision-making and representative of the population, although it has been observed that lower rates would be enough to have a protective effect.^13^ Expecting a high uptake would not be realistic if we think in groups of vulnerable people with no access to smartphones or with poor digital skills. The consequences complicate as we add AI components to the digital intermediation. For example, in AI-based tracing it is crucial to assess the accuracy of predictions and explain to the users where these come from so they can make sense of the messages received and actions they can take.

Unfortunately, the debates at the beginning of the pandemic triggered opposite positions that have distracted from the complexity of the problem and the need to set up long-term efforts dedicated to the study of similar scenarios. For example, the popular centralised vs. decentralised dispute, while opening important technical details to a broad audience, has implicitly encouraged the idea that a societal problem, like privacy and surveillance, can be fixed by a technical strategy (technical solutionism). Also, this and similar discussions hide the fact that privacy concerns are a prerogative also in human-driven tracing and should be supervised in all circumstances. While the debates on technical requirements and their privacy guarantees abounded clear evidence of the efficacy and the effectiveness of contact tracing apps are still missing and in need of an in-depth policy evaluation (see for example, technical flaws cited in [30]).

The challenges raised by this new technical mediation can be partially tackled by design and their larger effects are still poorly understood. As we suggested, to assess the effectiveness and the appropriateness of adopted solutions there are many aspects that should be considered, including the real performances and users’ understanding. In addition, it would be important to evaluate how tech solutions interact with existing apparatus such as the health care system and governmental bodies.

The problems surrounding contact tracing apps and similar technologies do not regard isolated efforts but encompass views and ideologies on how technology can serve society. All this needs a long-term discussion engaging different stakeholders, from health experts, engineers to politicians, and allowing citizens to actively contribute with feedback and comments. Similar work could be carried out by dedicated entities, like living labs or research hubs where both public and private institutions can collaborate to investigate possible scenarios and study the impact on society, ethical consequence and interaction with existing laws.

## Footnotes

1 Breaches have, for instance, been reported with regard to the sloppy roll-out of the NHS app in the UK. See: https://www.theguardian.com/technology/2020/jul/20/uk-government-admits-breaking-privacy-law-with-test-and-trace-contact-tracing-data-breaches-coronavirus

2 https://theconversation.com/contact-tracing-how-physicians-used-it-500-years-ago-to-control-the-bubonic-plague-139248

3 For example, see the tracker systems provided by the Ada Lovelace Institute (https://www.adalovelaceinstitute.org/our-work/identities-liberties/covid-19-digital-contact-tracing-tracker/), Privacy International (https://privacyinternational.org/examples/tracking-global-response-covid-19?field_location_region_locale_target_id=Italy+%28238%29&sort_by=field_date_value&sort_order=DESC) and the report exploring the European landscape delivered by AlgorithmWatch and Bertelsmann Stiftung (https://algorithmwatch.org/en/publication/new-report-on-adm-systems-in-the-covid19-pandemic/).

4 For instance, one may contend that the layer of automation introduced by notification apps is limited and does not justify their attribution to a machine-driven methodology. But, we believe that automated notifications have a serious impact on the whole tracing process, in particular with respect to the elicited behaviour. Indeed any notification is supposed to trigger a course of action involving the user, its contacts and the health authority.

5 Note that a machine-driven solution includes one or more (semi-)automated mechanisms but does not necessarily imply the use of Artificial Intelligence components.

6 Consider for example the app designed by Umea University https://simassocc.org/assocc-agent-based-social-simulation-of-the-coronavirus-crisis/the-reporting-app/# The aim of the application is “to collect information from Swedish inhabitants on their daily movements and overall situation. Reporting through the app is not based on any tracking or sensors but on the voluntary filling of a form.” In combination with an agent-based social simulation software, it provides information that can help governments and policy makers understand the effect of the policies that are being used to contain the spread of the virus.

7 Though GPS and network-based location tracking differ in accuracy and the way in which they gather location data, we consider them as part of the same class of location-based approach for the sake of simplicity.

8 See for example https://www.sondehealth.com/sondeone-page

9 According to the World Health Organization, contact tracing is “the process of identifying, assessing, and managing people who have been exposed to a disease to prevent onward transmission. When systematically applied, contact tracing will break the chains of transmission of an infectious disease and is thus an essential public health tool for controlling infectious disease outbreaks.” https://www.who.int/publications/i/item/contact-tracing-in-the-context-of-covid-19

10 Although this section focuses more on privacy and data protection, further considerations would include the right to health care and non-discrimination (see articles 21 and 35 of the Charter of Fundamental Rights of the European Union: https://eur-lex.europa.eu/legal-content/EN/TXT/?uri=CELEX:12012P/TXT).

11 See for example the Arrêté Royal n° 44 du 26 juin 2020, art. (14,§3,3°) in Belgium, the Ordonnance 818.101.25 sur le système de traçage de proximité pour le coronavirus SARS-CoV-2 du 24 juin 2020, art. 4 in Switzerland or the Décret n° 2020–650 du 29 mai 2020 relatif au traitement de données dénommé “StopCovid”, Art. 1 in France.

12 For an example of project studying citizen participation in technology assessment see: https://cordis.europa.eu/article/id/86647-participatory-technology-assessment

13 https://www.technologyreview.com/2020/06/05/1002775/covid-apps-effective-at-less-than-60-percent-download/
